# Baicalein Induces Apoptosis and Autophagy via Endoplasmic Reticulum Stress in Hepatocellular Carcinoma Cells

**DOI:** 10.1155/2014/732516

**Published:** 2014-06-03

**Authors:** Zhongxia Wang, Chunping Jiang, Weibo Chen, Guang Zhang, Dongjun Luo, Yin Cao, Junhua Wu, Yitao Ding, Baorui Liu

**Affiliations:** ^1^Department of Hepatobiliary Surgery, the Affiliated Drum Tower Hospital of Nanjing University Medical School, Nanjing, Jiangsu 210008, China; ^2^Department of Hepatobiliary Surgery, Nanjing Drum Tower Hospital Clinical College of Traditional Chinese and Western Medicine, Nanjing University of Chinese Medicine, Nanjing, Jiangsu 210008, China; ^3^Department of Hepatobiliary Surgery, Nanjing Drum Tower Hospital Clinical College of Nanjing Medical University, Nanjing, Jiangsu 210008, China; ^4^School of Medicine, Nanjing University, Nanjing, Jiangsu 210093, China; ^5^The Comprehensive Cancer Center, the Affiliated Drum Tower Hospital of Nanjing University Medical School, Nanjing, Jiangsu 210008, China; ^6^The Comprehensive Cancer Center, Nanjing Drum Tower Hospital Clinical College of Traditional Chinese and Western Medicine, Nanjing University of Chinese Medicine, Nanjing, Jiangsu 210008, China

## Abstract

*Background*. Hepatocellular carcinoma (HCC) remains a disastrous disease and the treatment for HCC is rather limited. Separation and identification of active compounds from traditionally used herbs in HCC treatment may shed light on novel therapeutic drugs for HCC. *Methods*. Cell viability and colony forming assay were conducted to determine anti-HCC activity. Morphology of cells and activity of caspases were analyzed. Antiapoptotic Bcl-2 family proteins and JNK were also examined. Levels of unfolded protein response (UPR) markers were determined and intracellular calcium was assayed. Small interfering RNAs (siRNAs) were used to investigate the role of UPR and autophagy in baicalein-induced cell death. *Results*. Among four studied flavonoids, only baicalein exhibited satisfactory inhibition of viability and colony formation of HCC cells within water-soluble concentration. Baicalein induced apoptosis via endoplasmic reticulum (ER) stress, possibly by downregulating prosurvival Bcl-2 family, increasing intracellular calcium, and activating JNK. CHOP was the executor of cell death during baicalein-induced ER stress while eIF2**α** and IRE1**α** played protective roles. Protective autophagy was also triggered by baicalein in HCC cells. *Conclusion*. Baicalein exhibits prominent anti-HCC activity. This flavonoid induces apoptosis and protective autophagy via ER stress. Combination of baicalein and autophagy inhibitors may represent a promising therapy against HCC.

## 1. Introduction

Hepatocellular carcinoma (HCC) represents a major health problem worldwide. It is the fifth most common cancer and ranks 3rd among the causes of cancer-related death [1]. Treatment of HCC largely relies on surgical resection, liver transplantation, and radiofrequency ablation, which are potentially curative interventions. However, a majority of HCC patients were diagnosed at advanced stage, especially in less-developed countries. For late-stage HCC, radical therapies are not suitable [2]. Options of treatment at this situation are even more limited. There is still no effective systemic chemotherapy available for HCC, which is notoriously known as a highly resistant cancer to most of the drugs [3]. Although transarterial chemoembolization (TACE) and orally available targeted drug sorafenib are proven to increase survival in selected candidates, the prognosis of advanced-stage HCC patients remains poor [4].

HCC often develops on the background of viral hepatitis, nonalcoholic fatty liver disease, alcoholic cirrhosis, and other sorts of chronic liver injury which ultimately transform hepatocytes to malignancies through oxidative stress, inflammation, and accumulation of mutations during injury-repair cycles [2, 4, 5]. Such circumstances may put endoplasmic reticulum (ER) under stress [6, 7]. To cope with ER stress, cells evoke an adaptive mechanism named unfolded protein response (UPR). Three ER transmembrane receptors, protein kinase R-like endoplasmic reticulum kinase (PERK), inositol-requiring enzyme 1 (IRE1), and activating transcription factor 6 (ATF6), initiate UPR through a signaling network. When UPR fails to rebuild homeostasis, programmed cell death could be induced to eliminate injured cells [8]. Along with UPR, autophagy could be triggered. The activation of autophagy flux reflects a possible compensatory reaction to relieve the burden of unfolded proteins and damaged organelles by autophagic degradation [9]. However, autophagy may either protect stressed cells or promote cell death via autophagic pathways. The fate of cells under ER stress might result from the balance between UPR and autophagy [10]. Growing evidence indicates the role of ER stress and autophagy in hepatocarcinogenesis [11, 12]. On the other hand, activation of ER stress and modification of autophagy activity may shed light on novel potential therapeutic approaches against HCC [13–15].

The root of* Scutellaria baicalensis* Georgi (Huang-qin in Chinese) has been broadly used in remedies for hepatitis, cirrhosis, jaundice, and HCC in traditional Chinese, Japanese, and Korean medicine [16]. Current analysis of active constituents of this herbal medicine revealed that flavonoids such as baicalein, baicalin, wogonin, and wogonoside are responsible for its liver protective activity [17]. To date, emerging studies suggest these flavonoids exhibit anti-HCC effects. Induction of apoptosis and cell cycle arrest and inhibition of migration and invasion by active compounds in* Scutellaria baicalensis* Georgi have been reported [16–22]. Detailed mechanisms of the inhibitory effects of flavonoids from* Scutellaria baicalensis* Georgi remain elusive. Possible molecular mechanisms include 12-lipoxygenase (12-LOX) [19], PI3K/Akt [18, 20], MEK/ERK [22, 23], and NF-*κ*B [24] transduction pathways. In this present study, we further investigated the potential inhibitory activity of HCC cells by four major flavonoid components of* Scutellaria baicalensis* Georgi: baicalein, baicalin, wogonin, and wogonoside. This study also revealed the roles of ER stress and autophagy in baicalein-induced HCC cell apoptosis.

## 2. Materials and Methods

### 2.1. Reagents

Baicalein (purity 98%), baicalin (purity 95%), wogonin (purity > 98%), wogonoside (purity > 95%), and tunicamycin were obtained from Sigma-Aldrich (St. Louis, MO). Cell counting kit-8 (CCK-8) was purchased from Dojindo Laboratories (Kumamoto, Japan). 2-(4-Amidinophenyl)-6-indolecarbamidine dihydrochloride (DAPI) and Fluo-3 AM were from Beyotime Institute of Biotechnology (Nantong, China). Antiphospho-PERK (Thr-981) rabbit polyclonal antibody (sc-32577) was purchased from Santa Cruz Biotechnology (Santa Cruz, CA). Other antibodies were obtained from Cell Signaling Technology (Beverly, MA).

### 2.2. Cell Culture

Human HCC cell lines SMMC-7721 and Bel-7402 were purchased from Cell Bank of Shanghai Institute of Biological Sciences, Chinese Academy of Sciences. SMMC-7721 cells were cultured in Dulbecco's modified Eagle's medium (DMEM, Gibco, Gaithersburg, MD) supplemented with 10% fetal bovine serum (10% FBS, Gibco, Gaithersburg, MD). Bel-7402 cells were maintained in RPMI-1640 medium (Gibco, Gaithersburg, MD) containing 10% FBS. All cell lines were maintained at 37°C in a humidified atmosphere with 5% CO_2_.

### 2.3. Cell Viability Evaluation

CCK-8 assay was used to evaluate relative cell viability. Briefly, 5 × 10^3^ cells growing on 96-well plate were treated with anticipated concentration of indicated flavonoids for 24 h or 48 h in triplicate. Control group was treated with dilution vehicle. After the desired time of treatment, medium with flavonoids was removed and 100 uL CCK-8 working solution diluted with fresh medium was added into each well. Cells were then incubated for another 4 h and optical density (OD) was measured at 450 nm using a VERSAmax microtiter plate reader (Molecular Devices Corporation, Sunnyvale, CA). Relative cell viability was calculated with the following formula: relative cell viability (%) = OD (treatment group)/OD (control group) × 100%.

### 2.4. Colony Forming Assay

300–500 cells were suspended in medium containing 10% FBS and plated in 6-well plates. After the attachment of cells for 24 h, they were treated with the indicated dose of flavonoids. After 24 h of treatment, fresh complete culture medium was changed and cell colonies were allowed to grow for 10 days. Colonies were then fixed with 3% paraformaldehyde and stained with 0.1% crystal violet for 30 min. Stained cell colonies were washed with phosphate buffered saline (PBS) for three times and dried. Images were obtained by a digital camera and colonies were counted using ImageJ software (U.S. National Institutes of Health, Bethesda, MD).

### 2.5. Western Blotting

Cell lysates were prepared by using radioimmune precipitation assay (RIPA) lysis buffer (Beyotime, Nantong, China) supplemented with a cocktail of protease inhibitors (Roche, Basel, Switzerland). Total protein concentration was determined by BCA reagent following the manufacturer's instruction (Thermo Scientific, Rockford, IL). Equal amounts of soluble proteins were separated by sodium dodecyl sulfate-polyacrylamide gel electrophoresis (SDS-PAGE). After being transferred to 0.45 *μ*m polyvinylidene difluoride (PVDF) membranes (Millipore, Bedford, MA), proteins were detected by incubation with primary antibodies followed by HRP-conjugated secondary antibodies. Enhanced chemiluminescence (ECL) reagent (Millipore, Bedford, MA) was applied to the membranes and specific protein bands were visualized by FluorChem FC2 Imaging System (Alpha Innotech, San Leandro, CA).

### 2.6. Fluorescence Microscopy Analysis

To determine the morphology of nuclei after drug treatment, cells were treated with or without the indicated concentration of baicalein for 24 h. Cells were then fixed with 3% paraformaldehyde and stained with 10 *μ*g/mL DAPI for 15 min. Images were captured with an Olympus BX53 fluorescence microscope (Olympus, Tokyo, Japan).

### 2.7. Measurement of Intracellular Calcium Concentration

Cells were treated with the indicated concentration of baicalein for 24 h before analysis. After the treatment, HCC cells were incubated with 5 *μ*M Fluo-3 AM calcium probe for 1 h. Medium containing Fluo-3 AM was then replaced by fresh medium and the cells were placed at 37°C for another 30 min to allow sufficient conversion of Fluo-3 AM into fluorescent Fluo-3. Cells were then detached by trypsin digestion and washed before detection of Fluo-3 on a FACSCalibur flow cytometer (BD Biosciences, San Jose, CA) following the manufacturer's instructions. Data were analyzed using FlowJo software (Treestar, Inc., San Carlos, CA).

### 2.8. Small Interfering RNA (siRNA) Transfection

siRNAs against human eIF2*α*, CHOP, IRE1*α*, Beclin 1, and Atg5 were synthesized by GenePharma (Shanghai, China). The sequences of siRNAs against eIf2*α*, CHOP, and IRE1*α* were from a previously published study by Shi et al. [25]. The sequences of other siRNAs were as follows: Atg5, GGGAAGCAGAACCAUACUATT; Beclin 1, CAGTTTGGCACAATCAATA. For transfection, SMMC-7721 cells were plated in 6-well plate and allowed to grow to 70% confluence. Transfection was conducted using Lipofectamine RNAiMAX reagent (Life Technologies, Carlsbad, CA) following the manufacturer's guidance. A scrambled siRNA was transfected as negative control.

### 2.9. Statistical Analysis

Numeric data were expressed as mean ± standard deviation (SD). Difference between groups was analyzed by one-way analysis of variance with Bonferroni's multiple comparisons. *P* < 0.05 was considered statistically significant.

## 3. Results

### 3.1. Baicalein Inhibits Proliferation of HCC Cells within Water-Soluble Concentrations

**Figure 1:**
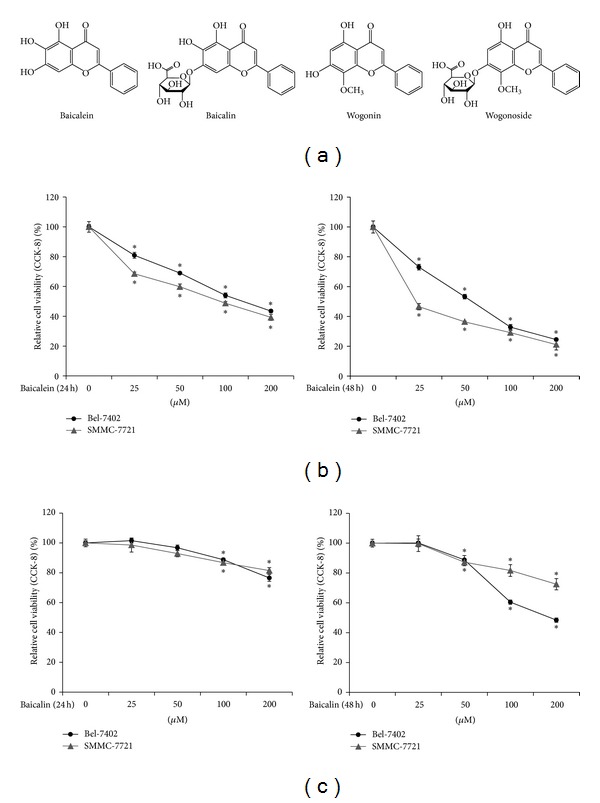
Baicalein inhibits proliferation of HCC cells. (a) Structures of the flavonoids used: baicalein, baicalin, wogonin, and wogonoside. (b) Human HCC cell lines Bel-7402 and SMMC-7721 were treated with 0, 25, 50, 100, and 200 *μ*M of baicalein for 24 h (upper panel) or 48 h (down panel). Relative cell viability was determined by CCK-8 assay. (c) Bel-7402 and SMMC-7721 cells were treated with 0, 25, 50, 100, and 200 *μ*M of baicalin for 24 h (upper panel) or 48 h (down panel). Relative cell viability was determined by CCK-8 assay. Data were expressed as mean ± SD. *P < 0.05, compared with control group.

We firstly undertook a study to preliminarily evaluate anti-HCC effects of four major flavonoids, baicalein, baicalin, wogonin, and wogonoside, from* Scutellaria baicalensis* Georgi. The structures of the compounds are shown in Figure 1(a). Two human HCC cell lines, SMMC-7721 and Bel-7402, were used for screening. The concentrations causing 50% inhibition of cell viability (IC_50_s) were listed in Table 1. After 24 h treatment, both baicalein and wogonin caused significant proliferation inhibition on HCC cells. In contrast, baicalin showed little activity against HCC cells with calculated IC_50_s markedly higher than baicalein in both cells. The effect of wogonoside on HCC cells was negligible. The proliferation of both SMMC-7721 and Bel-7402 cells remained uninterrupted even at 200 *μ*M concentration of wogonoside. We next prolonged the duration of drug treatment to further observe potential late effects of the tested flavonoids. Of note, the inhibitory effect of baicalein at 48 h increased dramatically whereas the IC_50_ values of wogonin only slightly dropped. At the same time, the IC_50_ of baicalin against Bel-7402 cells decreased to 169.35 *μ*M though the value for SMMC-7721 remains relatively high. Wogonoside showed no activity on both of the HCC cell lines even at 48 h.

In summary, our preliminary evaluation revealed that baicalein exhibited significant inhibition of proliferation of HCC cells in a time- and dose-dependent manner (Figure 1(b)). However, its glycoside baicalin showed only weak activity towards liver cancer cells (Figure 1(c)). On the other hand, although wogonin notably decreased the viability of HCC cells, its poor water solubility prevented us from further investigating this activity since this compound easily crystalized at lower concentration, especially when contrasted with the satisfactory solubility of baicalein within the wide testing concentration range. Even when treated with 200 *μ*M wogonoside for 48 h, proliferation of the tested cells remained intact, suggesting wogonoside had no inhibitory activity on HCC cells.

### 3.2. Baicalein Prevents HCC Cells from Forming Colonies

**Figure 2:**
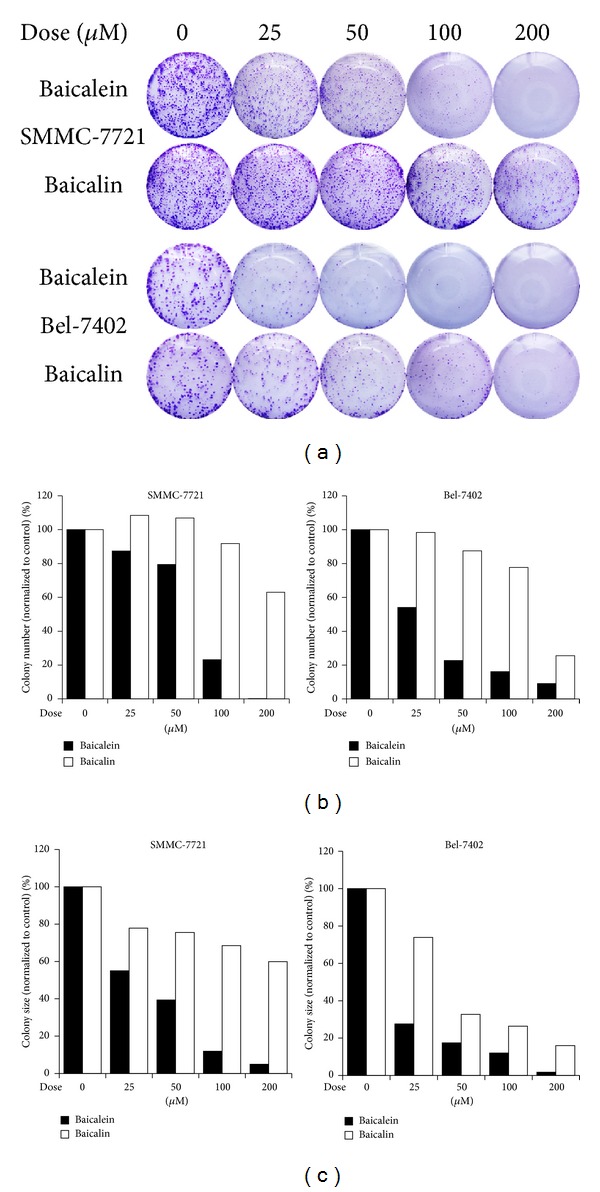
Baicalein inhibits colony formation of HCC cells. (a) SMMC-7721 and Bel-7402 cells were treated with the indicated dose of baicalein or baicalin. Cell colonies were visualized by crystal violet staining. (b) The amount of cell colonies formed after treatment of either baicalein or baicalin. Data were normalized to control and expressed as percentage. (c) The size of cell colonies after treatment of the indicated dose of baicalein or baicalin. Data were normalized to control and expressed as percentage.

To study the anti-HCC effect of baicalein, we conducted colony forming assay to observe if baicalein interferes with the ability of single cell to form growing colony, which represents an important character of cancer cells' ability to attach, survive, and proliferate. As shown in Figure 2(a), baicalein treatment dose-dependently suppressed the formation of HCC cell colonies in both SMMC-7721 and Bel-7402 cells. Similar to the results of cell viability assay, baicalin exhibited only a weak activity at higher doses against Bel-7402 cells. Measurements of colony number and colony size indicated that baicalein reduced both the amount and size of colonies in a dose-dependent manner. Interestingly, baicalin showed inhibition of foci size of Bel-7402 without an obvious decline of colony amount while its activity against SMMC-7721 cell colony formation remained minimal (Figures 2(b) and 2(c)).

### 3.3. Baicalein Induces Apoptosis in HCC Cells

**Figure 3:**
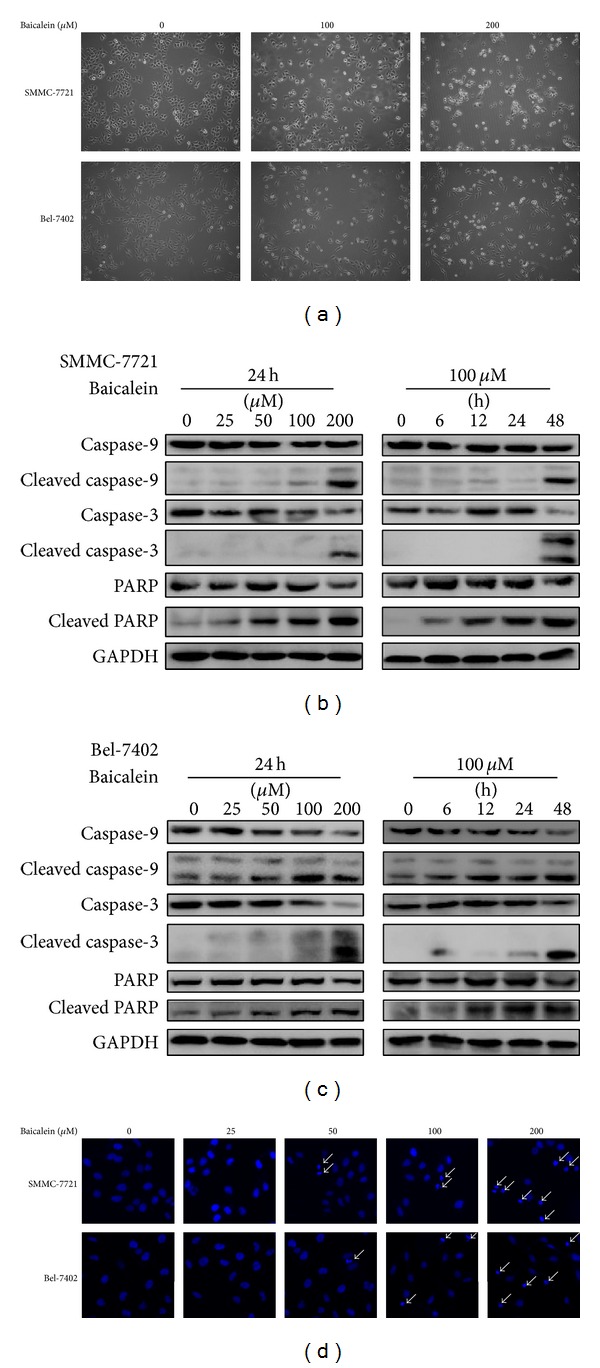
Baicalein induces apoptosis in HCC cells. (a) Morphology of SMMC-7721 and Bel-7402 cells under contrast microscopy (40x) after treating with 0, 100, or 200 *μ*M of Baicalein for 24 h. (b and c) The protein levels of full length and cleaved form of caspase-9, caspase-3, and PARP in SMMC-7721 (b) and Bel-7402 (c) cells were determined by western blotting following the treatment of the indicated dose of baicalein for the indicated time. GAPDH served as a loading control. (d) Morphology of nuclei after treatment of the indicated dose of baicalein for 24 h. Pyknosis and karyorrhexis were pointed by white arrow.

We next investigated the type of cell death underlying the inhibition of HCC cells mediated by baicalein. Following the treatment of baicalein, the appearance of HCC cells dramatically changed. As shown in Figure 3(a), cells in control group were in a typical polygonal or spindle-like intact appearance whereas baicalein-treated cells showed cell shrinkage, rounding, and blebbing and finally detached and floated in culture medium, which were representative morphological changes of apoptosis. To determine if cell death induced by baicalein was mediated by apoptosis, we examined the activity of caspase pathway by western blotting. The results indicated that baicalein caused marked cleavage of caspase-9, caspase-3, and PARP dose- and time-dependently. The induction of PARP cleavage happened as early as 12 h posttreatment (Figures 3(b) and 3(c)). The morphology of nuclei also showed typical appearances of apoptosis such as pyknosis and karyorrhexis (Figure 3(d)). Taken together, these results demonstrated that baicalein promoted HCC cell death through inducing apoptosis.

### 3.4. Baicalein Induces ER Stress and Activates UPR Pathways

**Figure 4:**
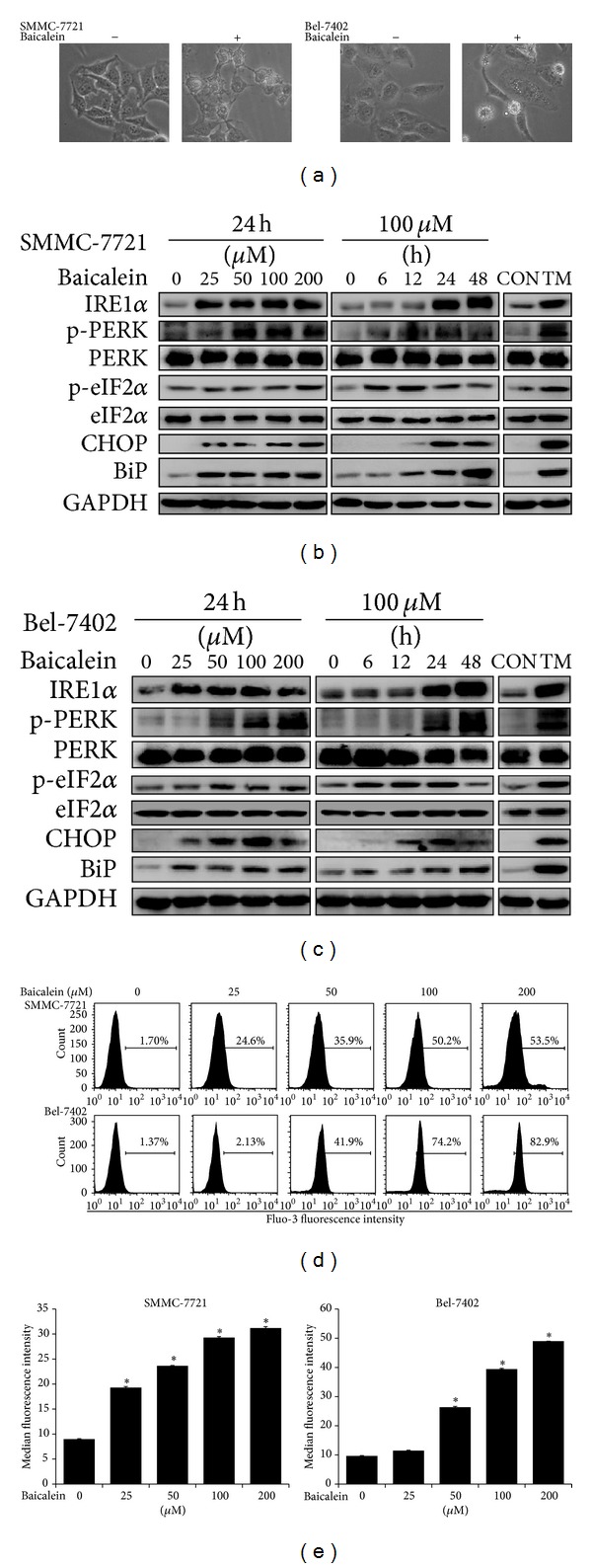
Baicalein induces ER stress. (a) Morphology change of HCC cells after the treatment of 100 *μ*M Baicalein (100x). (b and c) Levels of UPR proteins in SMMC-7721 (b) and Bel-7402 (c) cells were determined by western blotting after the treatment of the indicated dose of baicalein for the indicated time. Tunicamycin (TM, 5 *μ*g/mL) treatment for 6 h was used as positive control of ER stress induction. CON: control cells without drug treatment. GAPDH served as a loading control. (d) Intracellular calcium level of HCC cells was analyzed by flow cytometry. Cells were treated with the indicated concentration of baicalein for 24 h. (e) Median fluorescence intensity of calcium probe in HCC cells after treatment of the indicated dose of baicalein for 24 h. *P < 0.05, compared with control group.

During baicalein-induced apoptosis, cellular vacuolization was observed using contrast microscopy in dying cells while morphologically normal cells were free of this phenomenon (Figure 4(a)). Previous study indicates that these cytoplasmic vacuoles may be dilated ER lumens under stress [26]. We therefore conducted western blotting to determine whether baicalein-treated cells were under ER stress. As shown in Figures 4(b) and 4(c), PERK and IRE1*α*, receptors responsible for UPR signaling, were significantly activated dose- and time-dependently. Accordingly, the levels of several UPR downstream molecules such as CHOP and phosphorylated eIF2*α* were also upregulated at as early as 6 h and 12 h after baicalein treatment. As a responsive feedback, the expression of chaperone protein BiP was also enhanced. The expression patterns of these UPR-related proteins in baicalein-treated cells were consistent with cells treated by a well-characterized ER stress inducer, tunicamycin. Intracellular calcium homeostasis is among the functions of ER and aberrant calcium distribution may represent a typical manifestation of ER stress. Flow cytometry was employed to study intracellular calcium concentration using Fluo-3 AM calcium-sensitive fluorescence probe. Our results revealed that baicalein-induced prominent elevation of cytoplasmic calcium level (Figure 4(d)). The median fluorescence intensity of calcium probe escalated in a dose-dependent manner and reached as high as 3–5 times over vehicle control cells (Figure 4(e)). These results suggested that baicalein triggered ER stress in HCC cells and activated UPR signaling pathways, which may be closely related to apoptosis induced by this flavonoid.

### 3.5. Baicalein Suppresses the Expression of Antiapoptotic Bcl-2 Family Proteins and Activates JNK

**Figure 5:**
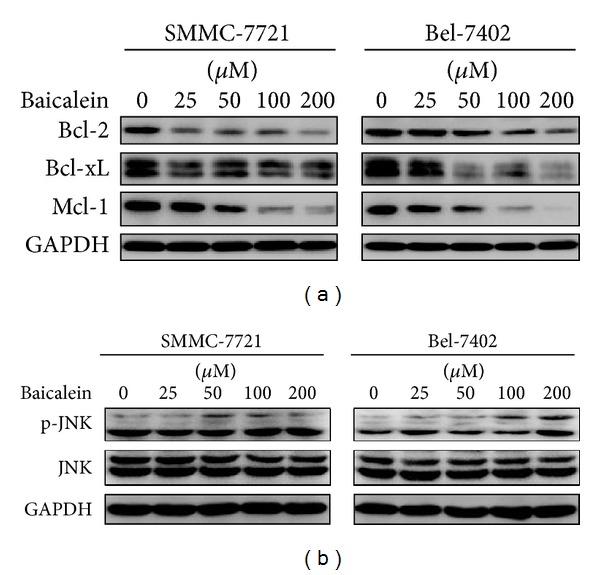
Baicalein suppresses the expression of antiapoptotic Bcl-2 family proteins and activates JNK pathway. (a) SMMC-7721 and Bel-7402 cells were treated with the indicated dose of baicalein for 24 h. Levels of Bcl-2, Bcl-xL, and Mcl-1 were determined by western blotting. (b) Phosphorylated JNK and total JNK were analyzed by western blotting after cells were treated with the indicated dose of baicalein. GAPDH served as a loading control.

It is reported that antiapoptotic Bcl-2 family proteins are downregulated during ER stress and JNK is activated to turn the balance towards apoptosis [10]. To test if this regulation also occurred when HCC cells were treated with baicalein, we studied the levels of Bcl-2, Bcl-xL, and Mcl-1, which are typical antiapoptotic Bcl-2 family members. As shown in Figure 5(a), baicalein suppressed the expression of these antiapoptotic regulators in both HCC cell lines. Meanwhile, phosphorylation of JNK was also detected in a dose-dependent manner, indicating that JNK pathway was activated after baicalein treatment (Figure 5(b)).

### 3.6. CHOP Induction Is Required for ER Stress-Mediated Apoptosis While eIF2*α* and IRE1*α* Play Protective Roles

**Figure 6:**
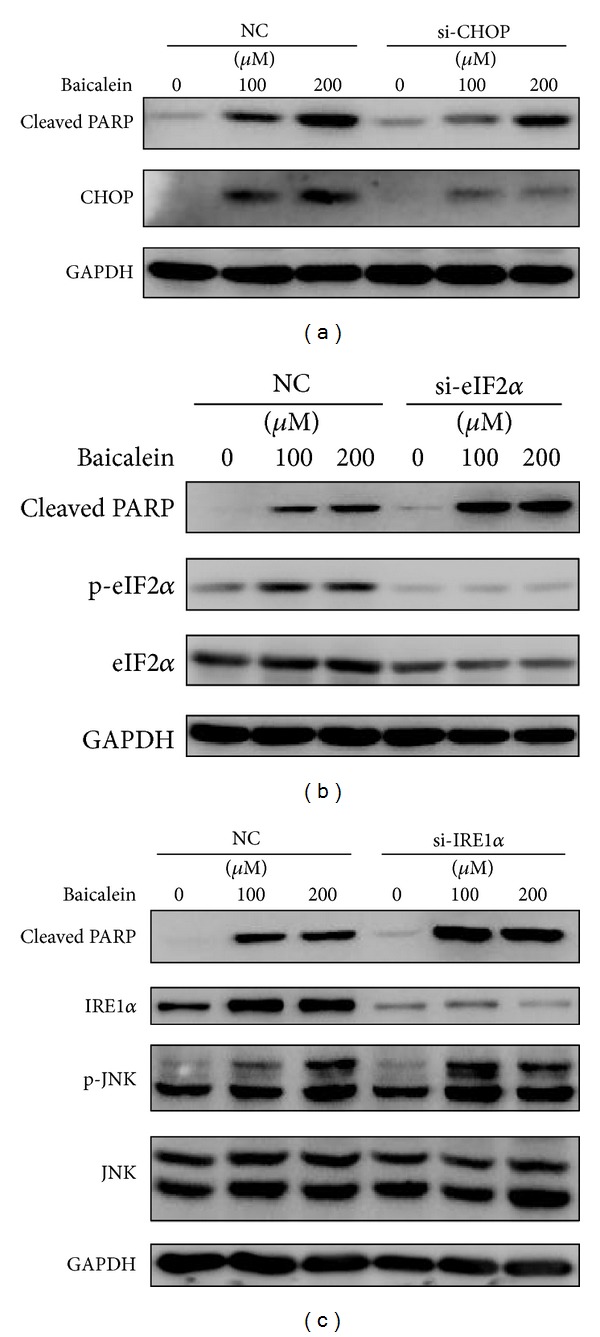
Diverse roles of UPR proteins in baicalein-induced apoptosis.(a) SMMC-7721 cells were transfected with scrambled RNA (NC) or CHOP-targeting siRNA (si-CHOP) for 48 h and treated with 0, 100, and 200 *μ*M baicalein for 24 h. Protein levels of cleaved PARP and CHOP were determined by western blotting. (b) SMMC-7721 cells were transfected with scrambled RNA (NC) or eIF2*α*-targeting siRNA (si-eIF2*α*) and then treated with 0, 100, and 200 *μ*M baicalein for 24 h. Protein levels of cleaved PARP phosphorylated eIF2*α* and eIF2*α* were determined. (c) After being transfected with scrambled RNA (NC) or IRE1*α*-targeting siRNA (si-IRE1*α*), SMMC-7721 cells were treated with the indicated dose of baicalein for 24 h and subjected to western blotting to analyze the level of cleaved PARP, IRE1*α*, phosphorylated JNK, and total JNK. GAPDH served as a loading control.

To further explore the roles of UPR signaling pathways in baicalein-induced apoptosis, we used siRNA-mediated gene knockdown to suppress the expression of UPR transducing molecules. Transfection of CHOP-targeting siRNA significantly attenuated the induction of CHOP after baicalein treatment. Interestingly, the suppression of CHOP markedly reduced cell apoptosis as indicated by reduced amount of cleaved PARP (Figure 6(a)). siRNA knockdown significantly reduced the level of eIF2*α* and almost totally abolished the phosphorylation of this protein. Interestingly, inhibition of eIF2*α* activation dramatically increased apoptosis (Figure 6(b)). Similar to eIF2*α*, siRNA-mediated silencing of IRE1*α* also blocked the activation of this pathway and exacerbated cell death by baicalein. Although IRE1*α* was thought to activate JNK pathway to facilitate apoptosis, our results demonstrated that knockdown of IRE1*α* did not inhibit baicalein-induced JNK activation (Figure 6(c)).

### 3.7. Protective Autophagy Is Induced by Baicalein

**Figure 7:**
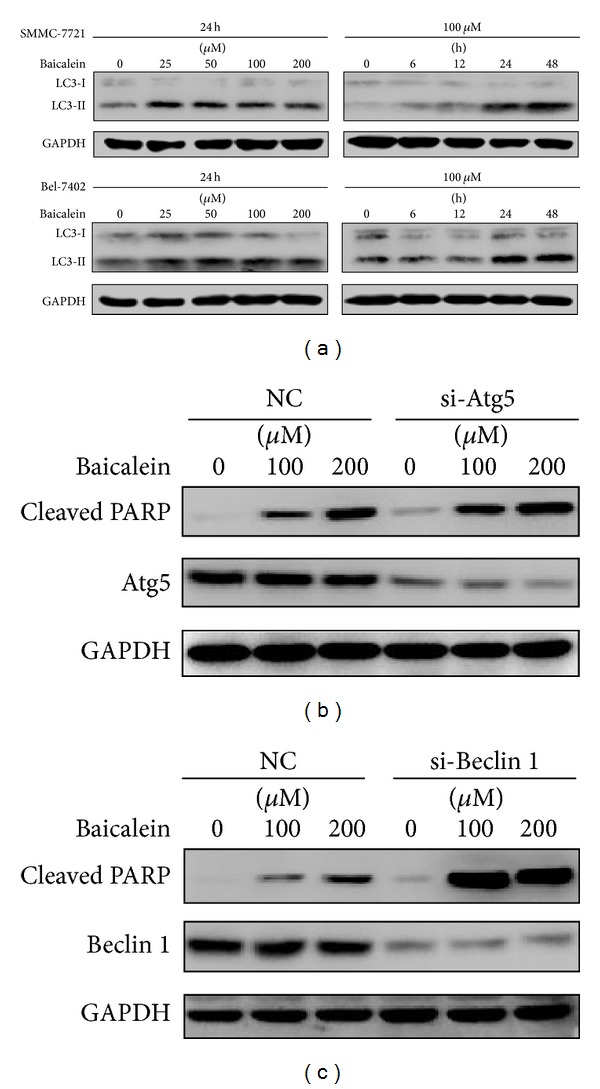
Baicalein induces protective autophagy. (a) HCC cells were treated with the indicated dose of baicalein for the indicated time and the level of LC-3 was determined. (b) SMMC-7721 cells were transfected with scrambled RNA (NC) or Atg5-targeting siRNA (si-Atg5) for 48 h and then treated with 0, 100, and 200 *μ*M baicalein for another 24 h. Cleaved PARP and Atg5 were analyzed by western blotting. (c) SMMC-7721 cells were transfected with scrambled RNA (NC) or Beclin 1-targeting siRNA (si-Beclin 1) for 48 h and incubated with the indicated concentration of baicalein for 24 h. Cleaved PARP and Beclin 1 were analyzed by western blotting. GAPDH served as a loading control.

We next investigated if baicalein induces autophagy, which is a frequently observed response coupling ER stress, in HCC cells. By western blotting, the conversion of LC-3I into LC-3II, a classic marker of autophagy activity, was determined. As shown in Figure 7(a), the amount of intracellular LC3-II was intriguingly increased in both tested cells, indicating possible upregulation of autophagy flux. To determine the role of baicalein-induced autophagy in cell death, we inhibited the expression of important regulators of autophagy pathway by siRNA. Our results showed that knockdown of Atg5 and Beclin 1 significantly aggravated apoptosis in baicalein-treated HCC cells (Figures 7(b) and 7(c)).

## 4. Discussion

In spite of recent advances in therapeutic strategies, HCC remains a disastrous disease for the majority of patients [27]. Surgical resection and liver transplantation are first-line treatments for HCC [4]. However, recurrence after surgery represents a tough problem and the prognosis of patients with recurrent disease is pessimistic [28]. For patients with advanced-stage HCC and without opportunity to receive curative therapy, effective treatment is even more limited [29]. HCC is well known for its resistance to chemotherapy. Systemic chemotherapy using traditional cytotoxic drugs has little effect on HCC patients; left small molecular targeted drug sorafenib is the only medication with evidence to improve prognosis of advanced-stage HCC [30, 31]. The absence of ideal therapy for HCC largely contributes to the current dilemma of HCC treatment. Therefore, much effort has been expended to discover novel molecular targets and potential effective drugs for HCC [32–34]. For thousands of years, herbal medicine had been widely used to treat liver diseases in China, Japan, Korea, and other districts around the world [35]. Separation and identification of active compounds from herbal medicine may provide potential drugs for HCC and help improve the prognosis of this deadly disease.

Huang-qin, the root of* Scutellaria baicalensis* Georgi, has been a major component of many traditional remedies for liver disorders, including HCC [17, 21, 36–38]. Modern sciences suggest that flavonoids in Huang-qin may be responsible for therapeutic effects of this herbal medicine [39]. In this study, we analyzed the inhibitory activity of four common flavonoids from Huang-qin (baicalein, baicalin, wogonin, and wogonoside) and found that baicalein showed potent inhibition of HCC cells within water-soluble concentration. This flavonoid also attenuated the ability of single HCC cell to form growing colony, which is an important character of cancer cells' ability to survive, attach, and proliferate to form tumors. Our results support several previous studies which reported the activity of baicalein against HCC cells [16–19, 22–24, 38, 40, 41]. This inhibition is of great importance because previous papers have provided evidence that baicalein preferentially kills HCC cells and leaves normal liver cells intact, demonstrating a selective anti-HCC activity [18, 23, 24].

However, the mechanisms of baicalein's anti-HCC activity remain elusive till now. Recent studies have shed light on potential molecular pathways involved in the activity of baicalein against HCC. Chang et al. revealed that baicalein induces cell cycle arrest and apoptosis in HCC cells [16]. Their later study indicated that apoptosis induced by baicalein may be attributed to mitochondrial dysfunction [17]. Mitochondria-dependent caspase pathway as well as AIF and Endo G pathways is also found to contribute to the induction of apoptosis by baicalein [41]. Our results also proved that cell death caused by baicalein is caspase-mediated apoptosis, supported by typical apoptotic morphology and change of nuclei appearance.

As for the role of signaling pathways in baicalein-induced HCC inhibition, Liang et al. recently revealed that MEK/ERK plays an important role both* in vitro* and* in vivo*. Baicalein inhibits MEK1 and subsequently reduces the activation of ERK1/2, leading to apoptosis and tumor growth arrest in mice bearing liver cancer [23]. Suppression of this pathway may also lead to attenuated cell migration and invasion by blocking multiple proteases degrading extracellular matrix [22]. The antitumor effect of baicalein may also be attributed to the deactivation of PI3K/Akt pathways. A recent study from Zheng et al. demonstrated that baicalein inhibited Akt and promoted the degradation of *β*-catenin and cyclin D1 independent of GSK-3*β*. This result is also confirmed in animal model [18]. Besides the abovementioned pathways, NF-*κ*B may also be responsible for the anticancer activity of baicalein [24].

Our present study provides additional mechanism explaining baicalein-induced HCC cell death. When observing the morphology of HCC cells undergoing apoptosis, we found an interesting phenomenon that baicalein treatment induced cellular vacuolization in HCC cell lines. This leads us to hypothesize that the vacuoles may be enlarged ERs under stress [25]. The following investigation revealed that baicalein treatment significantly activated UPR receptors PERK and IRE1*α*. As a result, downstream signal transduction molecules such as eIF2*α* and CHOP were also phosphorylated and induced, respectively. BiP, an ER chaperone which helps in protein folding and inhibits UPR in resting state, was also markedly upregulated, implying a feedback response towards baicalein-induced ER stress [42]. ER acts as a significant intracellular calcium pool and regulates calcium homeostasis. Calcium mobilization from ER into cytosol represents an emblematical event in response to various stimuli and has been implicated in the regulation of ER stress and UPR [25, 43]. Using a sensitive fluorescent probe, we found that intracellular calcium level was dramatically elevated following baicalein treatment. Taken together, our results suggest that baicalein induces ER stress in HCC cells and activates UPR.

UPR is a highly conserved cellular response aimed at reducing the burden of unfolded protein and restoring ER homeostasis. Multiple signaling pathways participate in UPR and functions diversely. Upon activation, PERK phosphorylates and activates eIF2*α*. As a translational regulator, eIF2*α* leads to a general translation block to reduce protein load in ER, thus preventing cells from overstress [44]. A set of genes including CHOP may escape this block and are translated with priority [45]. When UPR fails to relieve continuing pressure brought by ER stress, CHOP is found to mediate cell death and eliminate injured cells. CHOP signaling increases protein synthesis and exacerbates ER stress as well as downregulating antiapoptotic Bcl-2 family genes, which tip the balance towards cell apoptosis [10, 43]. IRE1*α* signaling pathway may also play an important role in ER stress-related apoptosis via potentiating PERK signaling and upregulating CHOP [46]. It is also reported to initiate cell death by activating JNK pathway [47]. In contrast, there is also evidence supporting a prosurvival role of IRE1*α* [48, 49]. Elevated intracellular calcium level may also contribute to apoptosis of cells under ER stress [50]. Our results indicated that prosurvival Bcl-2 family proteins, Bcl-2, Bcl-xL, and Mcl-1, were downregulated during baicalein-induced ER stress. Meanwhile, JNK was activated. Intracellular calcium level also escalated as mentioned above. As consequences of ER stress brought by baicalein, downregulation of antiapoptotic factors, increase of calcium concentration, and activation of proapoptotic JNK pathway may cooperate to execute apoptosis in HCC cells. In siRNA knockdown assays, as hypothesized, suppression of executor protein CHOP protected cells from apoptosis. However, interference of eIF2*α* potentiated baicalein-induced apoptosis, which could be explained by this protein's role of “burden reliever” in ER stress. Interestingly, our results suggested that inhibition of IRE1*α* also promoted HCC cell apoptosis. Knockdown of IRE1*α* did not alleviate the activation of JNK, indicating that IRE1*α* may not be responsible for regulating the activity of JNK pathway in baicalein-induced ER stress. In summary, CHOP is the major executor of ER stress-related apoptosis after treatment of baicalein, while eIF2*α* and IRE1*α* serve as protective factors.

In addition to the roles of UPR molecules in ER stress-related apoptosis, accumulating evidence suggests that autophagy may also closely interact with ER stress to determine cell fate [9, 10]. Autophagy may either protect cells from destruction or act as an inducer of cell death [25]. In this study, we observed a significant increase of conversion from LC-3I to LC-3II, which represents an important event during activation of autophagy. Inhibition of autophagy activity by siRNA-mediated gene knockdown of key regulators of autophagy, Atg5 and Beclin 1, revealed that autophagy induced by baicalein may be protective for cells against the pressure of ER stress. This may implicate a possible strategy to enhance the anti-HCC activity of baicalein by synchronously inhibiting autophagy.

In conclusion, to the best of our knowledge, our study for the first time provided evidence that baicalein induces apoptosis and autophagy via ER stress in HCC cells. Baicalein may represent a potential therapeutic drug with promising inhibitory activity against HCC. A combination of baicalein with inhibitors of autophagy may further enhance its anti-HCC effect.

## Figures and Tables

**Table 1:** IC_50_ values of baicalein, baicalin, wogonin, and wogonoside.

IC_50_ (*μ*M)	SMMC-7721		Bel-7402	
	24 h	48 h	24 h	48 h
Baicalein	94.84	19.89	134.81	59.52
Baicalin	1246.10	837.24	400.39	169.35
Wogonin	53.39	42.71	77.13	49.65
Wogonoside	N/I	N/I	N/I	N/I

IC_50_: concentration at which cells were inhibited by 50%; N/I: no inhibition.
